# Sempervirine Mediates Autophagy and Apoptosis *via* the Akt/mTOR Signaling Pathways in Glioma Cells

**DOI:** 10.3389/fphar.2021.770667

**Published:** 2021-11-30

**Authors:** Gaopan Li, Yuhuan Zhong, Wenyi Wang, Xiaokang Jia, Huaichang Zhu, Wenwen Jiang, Yu Song, Wen Xu, Shuisheng Wu

**Affiliations:** ^1^ College of Pharmacy, Fujian University of Traditional Chinese Medicine, Fuzhou, China; ^2^ Academy of Integrative Medicine, Fujian University of Traditional Chinese Medicine, Fuzhou, China; ^3^ Centre of Biomedical Research and Development, Fujian University of Traditional Chinese Medicine, Fuzhou, China

**Keywords:** sempervirine, apoptosis, auto-phagosome, glioma, cell cycle

## Abstract

The potential antitumor effects of sempervirine (SPV), an alkaloid compound derived from the traditional Chinese medicine *Gelsemium elegans* Benth., on different malignant tumors were described in detail. The impact of SPV on glioma cells and the basic atomic components remain uncertain. This study aimed to investigate the activity of SPV *in vitro* and *in vivo*. The effect of SPV on the growth of human glioma cells was determined to explore three aspects, namely, cell cycle, cell apoptosis, and autophagy. In this study, glioma cells, U251 and U87 cells, and one animal model were used. Cells were treated with SPV (0, 1, 4, and 8 μM) for 48 h. The cell viability, cell cycle, apoptosis rate and autophagic flux were examined. Cell cycle, apoptotic, autophagy, and Akt/mTOR signal pathway-related proteins, such as CDK1, Cyclin B1, Beclin-1, p62, LC3, AKT, and mTOR were investigated by Western blot approach. As a result, cells induced by SPV led to G2/M phase arrest and apoptosis. SPV also promoted the effect of autophagic flux and accumulation of LC3B. SPV reduced the expression of p62 protein and induced the autophagic death of glioma cells. Furthermore, SPV downregulated the expressions of AKT and mTOR phosphorylated proteins in the mTOR signaling pathway, thereby affecting the onset of apoptosis and autophagy in U251 cells. In conclusion, SPV induced cellular G2/M phase arrest and blockade of the Akt/mTOR signaling pathway, thereby triggering apoptosis and cellular autophagy. The *in vivo* and *in vitro* studies confirmed that SPV inhibits the growth of glioma cancer.

## Introduction

Malignant primary brain tumours remain among the most difficult cancers to treat, with a 5 year overall survival no greater than 35% (Lapointe et al., 2018). They are characterized by high incidence, high fatality rate, and low cure rate (Sathornsumetee et al., 2007). Currently, temozolomide (TMZ) is the first-line chemotherapeutic drug for glioma treatment with a definite curative effect. However, the efficacy of TMZ is frequently limited by the durability of the chemoresistance response. Therefore, research that facilitates the development of innovative drugs is urgently needed.

*Gelsemiu* *m elegans* Benth., a flowering plant, is a world-famous poisonous plant distributed in Fujian, Yunnan, Guizhou, Guangxi, and other provinces of China. It is also distributed in southeast Asia (Jin et al., 2014). It has been used in traditional Chinese medicine to treat certain types of skin ulcer, cerebral pain, and cancer (Editorial Committee of Zhonghua Bencao National Traditional Chinese Herb Administration, 1999.). However, its clinical application was limited by its great toxicity.

However, sempervirine, which has trace amounts of yohimbane alkaloid, was separated from the *G. elegans* Benth. in 1949; its structure and chemical properties have been reported by Nature and J AM CHEM SOC (Goutarel et al., 1948; Bentley and Stevens, 1949; Woodward and Witkop, 1949; Ska, 2006). Since 2016, J AM CHEM SOC and other journals reported its synthesis and chemical structure modification (Pan et al., 2016; Sucunza et al., 2016; Kerkovius and Kerr, 2018). Previous research showed that sempervirine can distinguish between cancer and normal DNAs, and it can inhibit YC8 lymphoma cells and Ehrlich ascites carcinoma cells *in vitro* (Beljanski and Beljanski, 1986). Other research works showed that sempervirine is cytotoxic to human breast cancer MDA-MB-231, cervical cancer HeLa, and lymphoma Raji cells (Pan et al., 2016). In testicular germ cell tumors, sempervirine induces cell death by inhibiting RNA polymerase I transcription independently from p53 (Caggiano et al., 2020). In our previous study, normal cells (3T3-L1 and THP-1 cells) were treated with sempervirine at 1–8 μM, and no cytotoxicity was found. Mice still survived after treatment with 2 g∙kg^−1^ of sempervirine through intragastric administration. In addition, sempervirine can attack tumor cells but not normal cells through RPA194 (Caggiano et al., 2020). This finding suggests that sempervirine may be less toxic. Sempervirine may readily cross the blood brain barrier because of its small molecular weight, which may be advantageous in the treatment of intracranial tumors.

The smooth operation of tumor cell cycle is a necessary condition for cell proliferation, and the occurrence and development of tumors are positively/negatively correlated with the dysregulation of cell growth cycle regulatory proteins (Meroni et al., 2019). Glioma cells showed abnormal circulation and proliferation like other highly malignant solid tumor (Vitovcova et al., 2020). Many cytotoxic agents involve G2/M check point arrest inhibition by affecting the cyclin-CDK complex or inducing DNA damage, mitotic defects, and cytokinesis failure (Castro-Gamero et al., 2018).

Programmed cell death (PCD) of tumor cells under the control of certain genes and protein signaling pathways is among the mechanisms affecting glioma occurrence and development. Apoptosis and autophagy have been main research topics in recent years. Type I PCD apoptosis signaling pathways can be roughly divided into two ways, namely, endogenous mitochondria (way) and exogenous (death receptor pathway). Cell apoptosis signal first activates the mitochondrial pathway, that is, through the activation of apoptosis regulation factors (e.g., Bax) and regulatory factors (such as the Bcl2) of resistance to apoptosis gathered in the mitochondrial membrane. Then, mitochondrial cytochrome C release is stimulated, thereby changing the mitochondrial membrane potential to start the apoptosis; ultimately, molecular caspase activates downstream effect, caspase 3 is activated, and apoptosis is triggered after being induced by the caspase cascade (Van Opdenbosch and Lamkanfi, 2019; Rajabi et al., 2021). This PCD type is called II PCD, cell autophagy; along with apoptosis, it can regulate cell death (Chmurska et al., 2021). Hence, research needs to focus on the conceivable component fundamental sempervirine-initiated apoptosis or autophagy in glioma cells.

However, the mechanisms underlying the regulation of tumor genesis and development are complex, and the biological effects of tumor cell proliferation, apoptosis, and autophagy are strictly regulated by relevant signaling pathways. Among the pathways, the mTOR signaling pathway is closely related to the abovementioned mechanisms, and the activation of its signaling pathway is related to the expression of PI3K/Akt and other proteins (Li et al., 2016; FranziskaMarquard, 2019; Shahcheraghi et al., 2020). The signal pathways have interactive control and regulate the expression of Cyclin B1 and CDK1 proliferation-related factors, contain the Bcl-2 and Bax regulatory elements, and have specific protein autophagy regulation (LC3-II, Beclin 1, and p62 closely) (D Arcy, 2019).

We evaluated the antitumor activity of the total alkaloids and each monomer of *G. elegans* Benth and found that sempervirine can significantly inhibit human glioma cells. In the research, we pointed out the possible mechanisms of sempervirine effects and antitumor effects on glioma cells *in vitro* and *in vivo*. Sempervirine induced G2/M phase arrest in glioma cells and initiated apoptosis and autophagy by regulating the Akt/mTOR signaling pathway. In an extension, we found that sempervirine-induced autophagy can induce cell death.

## Materials and Methods

### Chemicals and Reagents

The chemicals used in our experiments were: Sempervirine (Percent Purity, ≥98.0%; ChemFaces, CFS201902), MTT (Sigma-Aldrich, M2128), z-VAD-FMK (Apexbio, A1902), 3-Methyladenine (3-MA, Apexbio A8353R), Rapamycin (Rap, Apexbio, A8167), MK2206 (MK, Beyotime, SF2712). The antibodies used in our experiments were: anti-CDK1 antibody (Proteintech, 19532-1-AP), anti-Cyclin B1 antibody (Proteintech, 55004-1-AP), anti-Bax antibody (Proteintech, 50599-2-lg), anti- Bcl-2 antibody (Proteintech, 12789-1-AP), anti-Caspase-3 antibody (Proteintech, 19677-1-AP), anti-cleaved-caspase-3 antibody (CST, 9661S), anti-LC3 antibody (CST, 12741S), anti-Beclin 1 antibody (Proteintech, 11306-1-AP), anti-p62 antibody (Proteintech, 18420-1-AP), anti-AKT antibody (Proteintech, 60203-2-Ig), anti-Phospho-Akt (Ser473) antibody (CST, 4060S), anti-mTOR antibody (CST, 28273-1-AP), anti-Phospho-mTOR antibody (CST, 5536T), anti-β-Actin antibody (Proteintech, HRP-60008).

### Cell Culture

The human glioma cell lines U251 and U87 were obtained from Chinese Authenticated Collection of Type Culture. The cell culture in DMEM and MEM (containing NEAA) (HyClone, United States) contained 10% fetal bovine serum (Gibco, United States) in 5% CO_2_ at 37°C.

### Cell Viability Assay

Cells in logarithmic growth phase (5 × 10^3^ cells per well) were inoculated into 96-well plates and incubated overnight. The next day, after aspirating the medium, cells were incubated for 24, 48, and 72 h in a medium containing different concentrations of sempervirine. Sempervirine was dissolved in DMSO and then diluted with cell culture medium. The final concentration of DMSO was much less than 0.1% (v/v) in all experiments. Cells treated with the same amount of DMSO were served as controls. Cell viability was measured by incubating cells with 10 μL of MTT for 4 h under light-free conditions. Absorbance was measured by a SpectraMax M5 microplate reader at 490 nm (Molecular Devices, United States).

### Clone Formation Assay

Cells were counted and then inoculated in 6-well plates at a density of 500–1,000 cells/well. After 24 h, the cells were treated with different concentrations of sempervirine (1, 4, and 8 μM) for 48 h and then left untreated. The medium was discarded after 14 days of incubation, and the cells were washed thrice with PBS. Cells were stained with 0.1% crystalline violet for 15 min. Clones were counted using Image Quant TL 7.0 software.

### Cell Cycle Analysis

After induction treatments with sempervirine at 0, 1, 4, and 8 μM, cells were precipitated with cold ethanol and then stained with propidium iodide (Beyotime, China) for 15 min. Individual cell nuclei (about 20,000 events) were analyzed for fluorescence by flow cytometry (BD, United States).

### Annexin V-FITC/PI Staining

Cells were inoculated into 6-well plates at a density of 3 × 10^5^ cells/well and incubated with different concentrations of sempervirine (0, 1, 4, and 8 μM) for 48 h. Cells were then washed, collected, processed according to the instructions of the Annexin V-FITC/PI Apoptosis Detection Kit, and analyzed by flow cytometry (BD, United States).

### Immunofluorescence Staining

The cells were fixed with cold 4% paraformaldehyde for 15 min and subjected to permeabilization in 0.3% Triton X-100. Then, after being blocked in 5% BSA for 1 h, the cells were incubated with primary antibodies against NLRP3 (1:100) and LC3A/B (1:100) overnight at 4°C. Then, the cells were incubated with the appropriate secondary antibody for 1.5 h at room temperature. DAPI (Sigma, United States) was used to stain the nucleus, and florescence images were captured by using a fluorescence microscope.

### Human Glioma Nude Mice Xenografted Experiment

All animal experiments were performed in strict accordance with the regulations of the Biomedical Ethics Committee of Fujian Medical University (Fujian, China). SPF-grade male BALB/c nude mice (18–20 g weight; 6 weeks old) were provided by SLAC Laboratory Animal Company. The conditions were as follows: cage temperature was controlled at 22°C–25°C; humidity was at 50–60%; 12 h light/12 h dark alternation; and free access to water and food.

U251 cells (5 × 10^6^ cells) were suspended in PBS and subcutaneously implanted in the right axilla of the nude mice. When the tumor size reached 90–100 mm^3^, the mice were randomly divided into four groups (n = 5). Then the four groups of mice were treated with vehicle (v/v) (1% tween 80, 2% DMSO, 97% physiological saline), 1 mg/kg sempervirine, 4 mg/kg sempervirine, and 8 mg/kg sempervirine every day by intraperitoneal injection, respectively. The tumor growth of U251 cells was inhibited for a total of 28 days. During drug injection, the body weight of each mouse was recorded, the tumor size was measured, and the tumor volume was calculated as (a^2^ × b)/2, where a represents the minimum diameter of the tumor, and b represents the maximum diameter of the tumor. All mice were cervically dislocated and sacrificed after treatment. The tumors were removed and weighed, the relative tumor weight was calculated and the inhibition rate was calculated according to following formulas:

$$\text{the relative tumor weight (\%) =}\frac{tumor\;weight}{animal\;weight}\text{×100\%}$$ (1)

$$\text{Inhibition ratio (\%) =}\frac{relative\;W_{c}-relative\;W_{t}}{relative\;W_{c}}\text{×100\%}$$ (2)

where relative W_c_ and relative W_t_ denote the mean relative tumor weight in the control and treatment groups, respectively.

### Western Blot Assay

After intervention of cells with different concentrations of sempervirine for 48 h, total cellular protein was extracted by adding RIPA and protein sample concentrations were detected. Approximately 20 μg of protein was loaded onto SDS-PAGE gel lanes and then was transferred to PVDF membranes. After sealing with 5% skim milk, membranes are incubated overnight at 4°C with primary antibody and then incubated for 2 h at room temperature with HRP-coupled secondary antibody. The immunoreactive staining was performed employing a chemiluminescence kit and after that visualized utilizing the Bio-Rad ChemiDoc XRS + System.

### Statistical Analysis

Experiments were performed thrice independently. Results were expressed as the mean ± SD. Multiple comparisons were performed using one-way ANOVA. All information was analyzed utilizing GraphPad Prism version 7.0 software (San Diego, CA, United States). Statistical significance was expressed as *p* < 0.05.

## Results

### Sempervirine Inhibits Cell Viability

**Figure 1:**
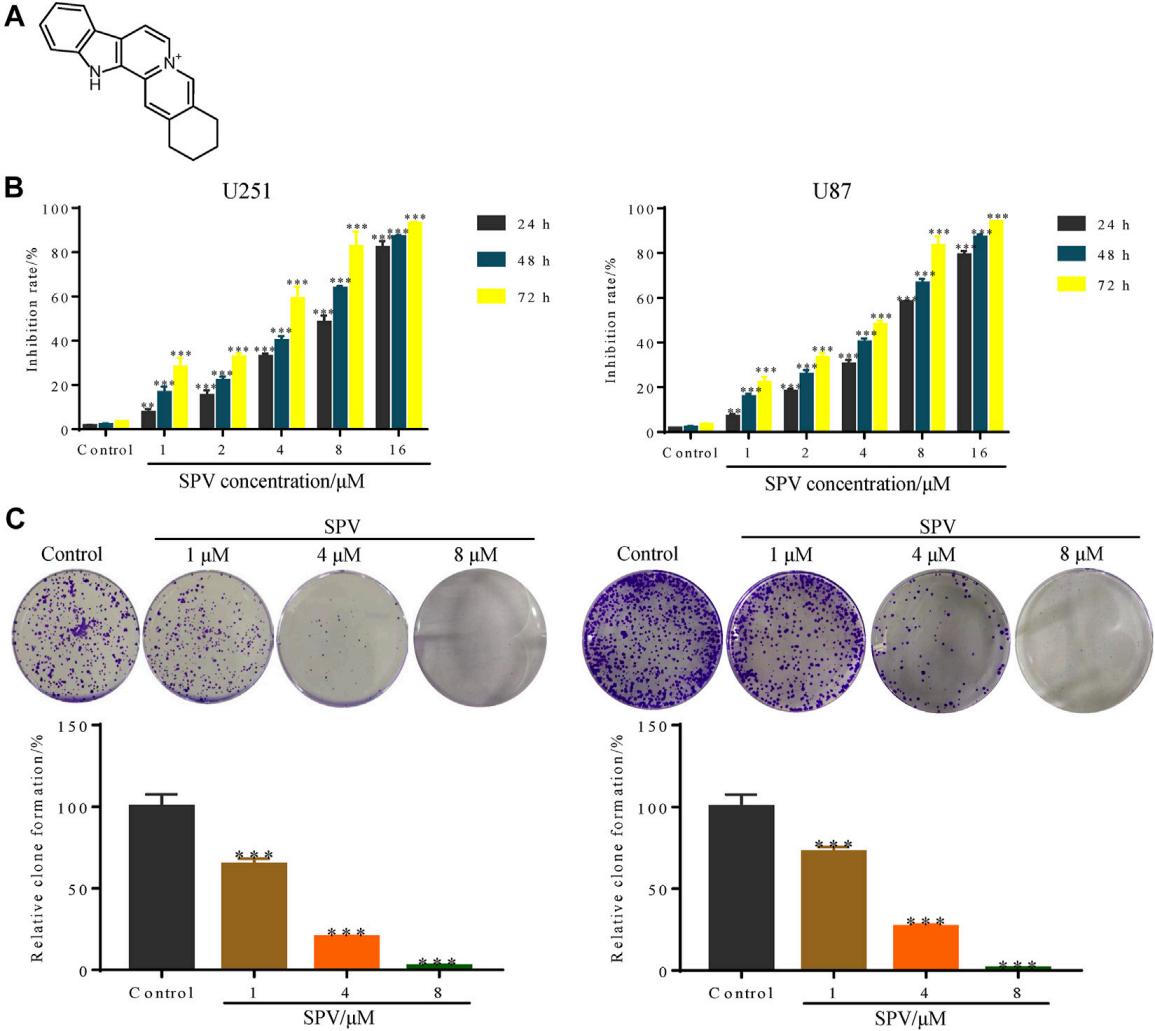
Sempervirine (SPV) inhibited cell viability in glioma cells. **(A)** The molecular structure of sempervirine. **(B)** Two glioma cell lines (U251, U87) were treated with sempervirine (0, 1, 2, 4, 8 and 16 μM) for 24, 48 and 72 h. Cell viability was measured by MTT assay. **(C)** U251 and U87 cells were treated with sempervirine (0, 1, 4 and 8 μM) for 48 h, and then were untreated for approximately 14 days. Cell colony formation was assessed. Data are presented as the Mean ± *SD* (*n* = 3). **p < 0.01 and ***p < 0.001 compared with the control group.

The chemical structure of sempervirine is shown in (Figure 1A). MTT results showed that different concentrations of sempervirine (0, 1, 2, 4, 8, and 16 μmol∙L^−1^) significantly reduced cell viability and inhibited cell colony formation in U251 and U87 cells, and the inhibitory effects of sempervirine on glioma cell proliferation were dose-dependent (Figures 1B,C). We evaluated the cell viability at 24, 48, and 72 h to determine the effect of sempervirine on cells and found that the IC50 of sempervirine at 48 h was approximately 4.981 ± 0.23 and 4.709 ± 0.095 μM for U251 and U87 cells, respectively.

### Sempervirine Induces Cell Cycle Arrest

**Figure 2:**
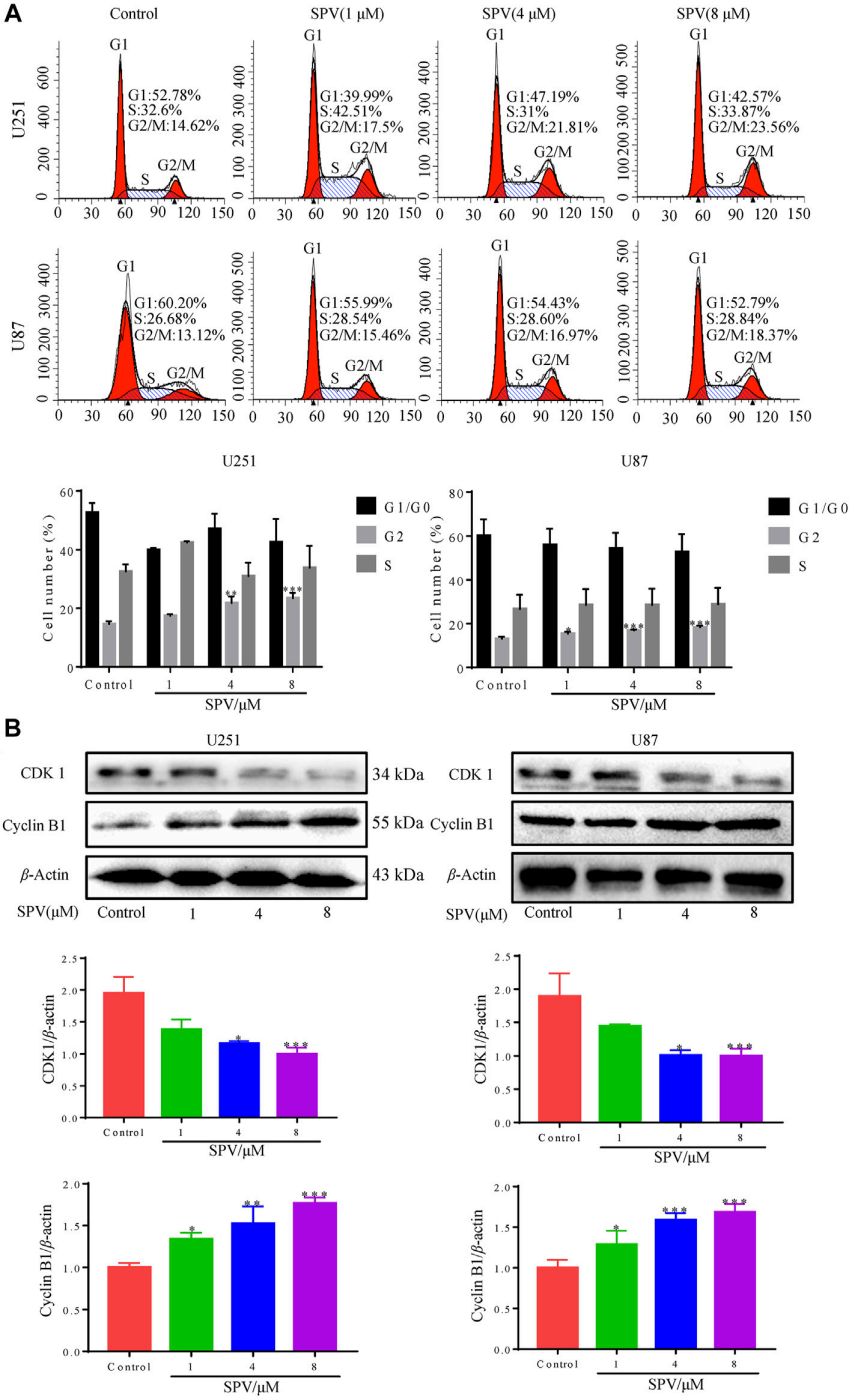
Sempervirine induced G2/M cell cycle arrest in glioma cells. **(A)** Cells were treated with sempervirine (0, 1, 4 and 8 μM) for 48 h and cell cycle distribution was analyzed by flow cytometry. **(B)** U251 and U87 cells were treated with sempervirine (0, 1, 4 and 8 μM) for 48 h. The levels of cell cycle-related protein expressions were determined by Western blotting. Data are presented as the Mean ± *SD* (*n* = 3). *p < 0.05, **p < 0.01 and ***p < 0.001 compared with the control group.

Cell cycle arrest can lead to proliferation inhibition. Therefore, we also examined the cell cycle profile of sempervirine-treated cells by flow cytometry (Figure 2A). The cells treated with 1, 4, and 8 μM sempervirine showed cell cycle arrest; the cells stayed in the G2/M phase. Next, we determined the expression of cell cycle-related protein CDK1 and cyclin B1 protein by Western blot assay. The expression levels of Cyclin B1 protein increased, and CDK1 protein expression decreased after treatment with sempervirine at concentrations of 4 and 8 μM, indicating that sempervirine caused G2/M cell cycle arrest, as shown in (Figure 2B).

### Sempervirine Induces Apoptosis

**Figure 3:**
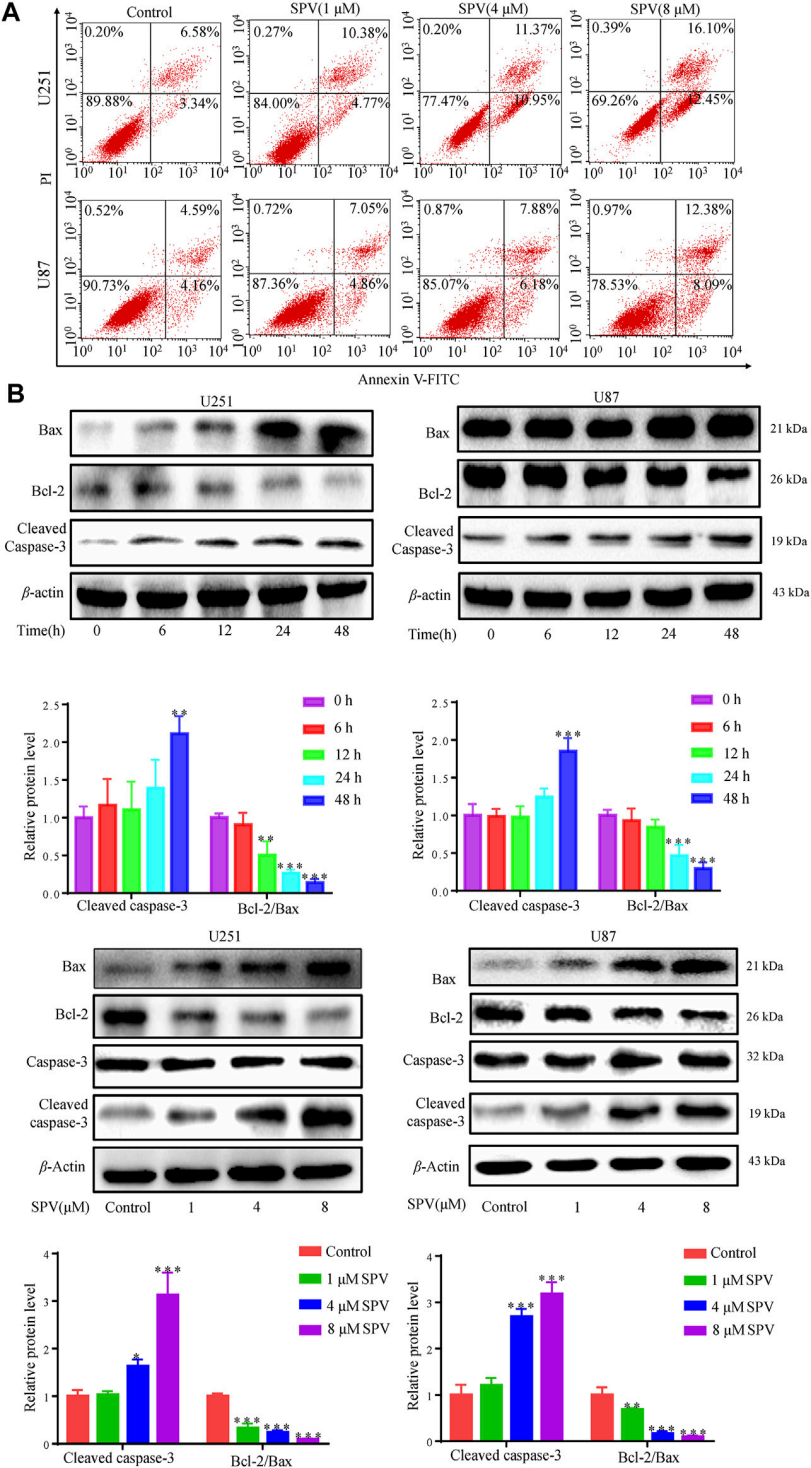
Sempervirine induced apoptosis in glioma cells. **(A)** Cells were treated with sempervirine (0, 1, 4 and 8 μM) for 48 h, apoptotic cells were analyzed by flow cytometry. **(B)** Cells were incubated with sempervirine (4 μM) for different durations or treated with various concentrations of sempervirine for 48 h. The apoptosis-related proteins bax, bcl-2, Caspase-3 and cleaved caspase-3 were determined by Western blotting. Data are presented as the Mean ± *SD* (*n* = 3). *p < 0.05, **p < 0.01 and ***p < 0.001 compared with the control group.

We examined the effect of sempervirine on apoptosis in U251 and U87 cells to investigate whether sempervirine also causes apoptosis. First, we assessed the apoptotic effect of sempervirine on cells by analyzing the apoptosis rate using the Annexin V/PI assay (Figure 3A). At sempervirine concentrations above 1 μM, the apoptotic rate of cells increased significantly in a time- and dose-dependent manner.

We focused on the apoptosis factors, such as, bcl-2, bax, and caspase 3. Sempervirine treatment increased the expression of cleaved caspase 3 and bax, whereas the anti-apoptotic protein bcl-2 was down-regulated in a dose- and time-dependent manner in GBM cells, as shown in (Figure 3B). Cell cycle arrest and increased activity of the early apoptotic protein caspase-3 after sempervirine treatment suggested that these cells entered the apoptotic pathway.

**Figure 4:**
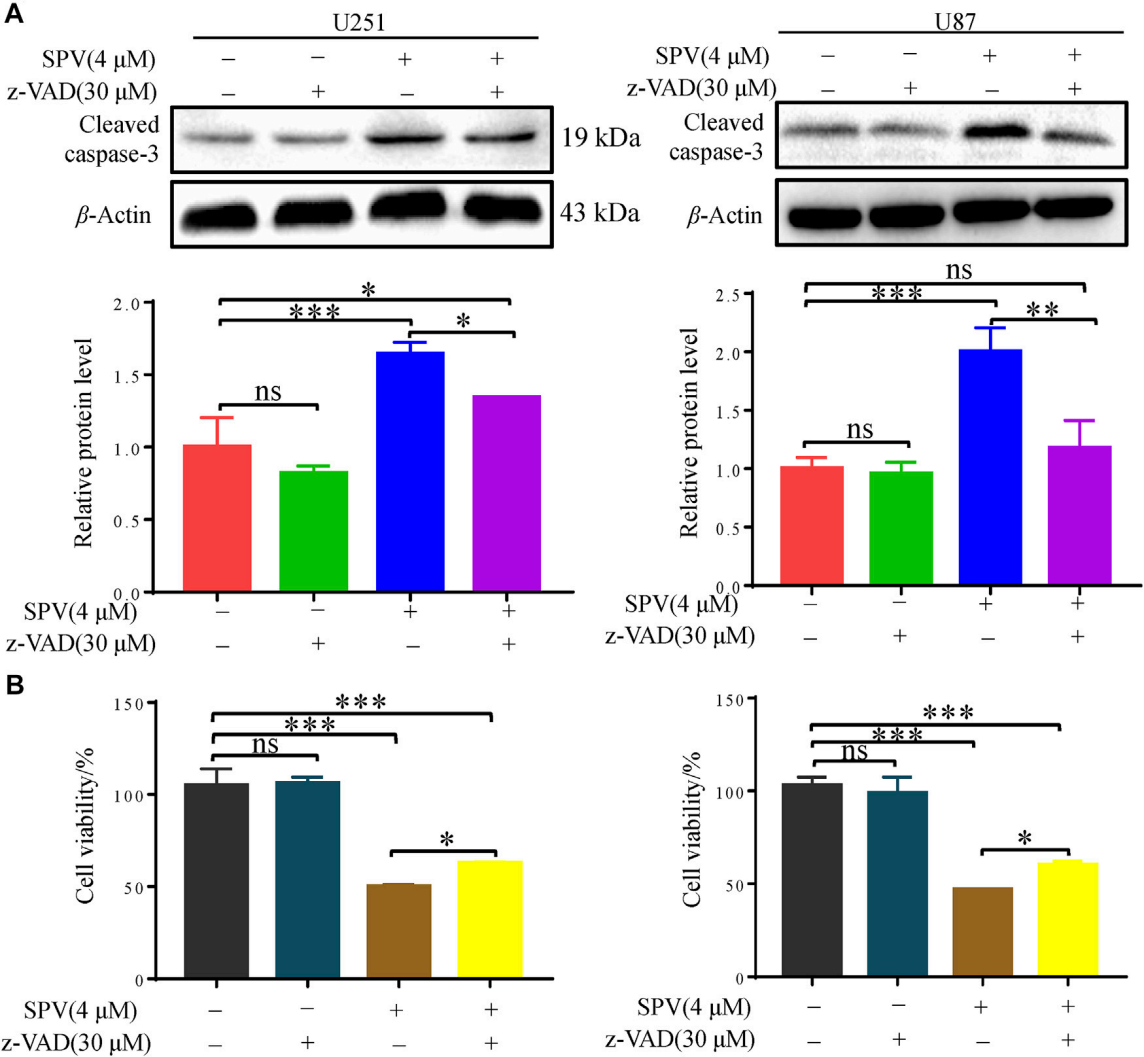
Sempervirine triggered apoptosis in glioma cells, and inhibition of apoptosis decreased sempervirine-induced cell death. **(A)** z-VAD (30 μM) was added to cells 2 h before sempervirine treatment. After 48 h, cleaved caspase-3 was analyzed by Western blotting. **(B)** z-VAD was added to cells 2 h before sempervirine treatment. Then, cells treated with sempervirine for 48 h. Cell viability was determined by MTT. Data are presented as the Mean ± *SD* (*n* = 3). *p < 0.05, **p < 0.01 and ***p < 0.001 compared with the control group.

Finally, we applied the apoptosis inhibitor (z-VAD-FMK) to U251 and U87 cells, and the changes in cell viability were analyzed. When cells were co-treated with z-VAD-FMK and sempervirine, z-VAD-FMK reversed the sempervirine-induced increase in the expression level of cleaved caspase 3 in the Western blot results (Figure 4A). No reduction in cell viability was found after the use of z-VAD-FMK alone on cells. However, compared with the group treated with sempervirine, co-incubation of cells with sempervirine and z-VAD-FMK increased U251 cell survival (Figure 4B). These results confirmed that sempervirine indeed induced the apoptosis of U251 cells.

### Sempervirine Induces Autophagy in Glioma Cells

**Figure 5:**
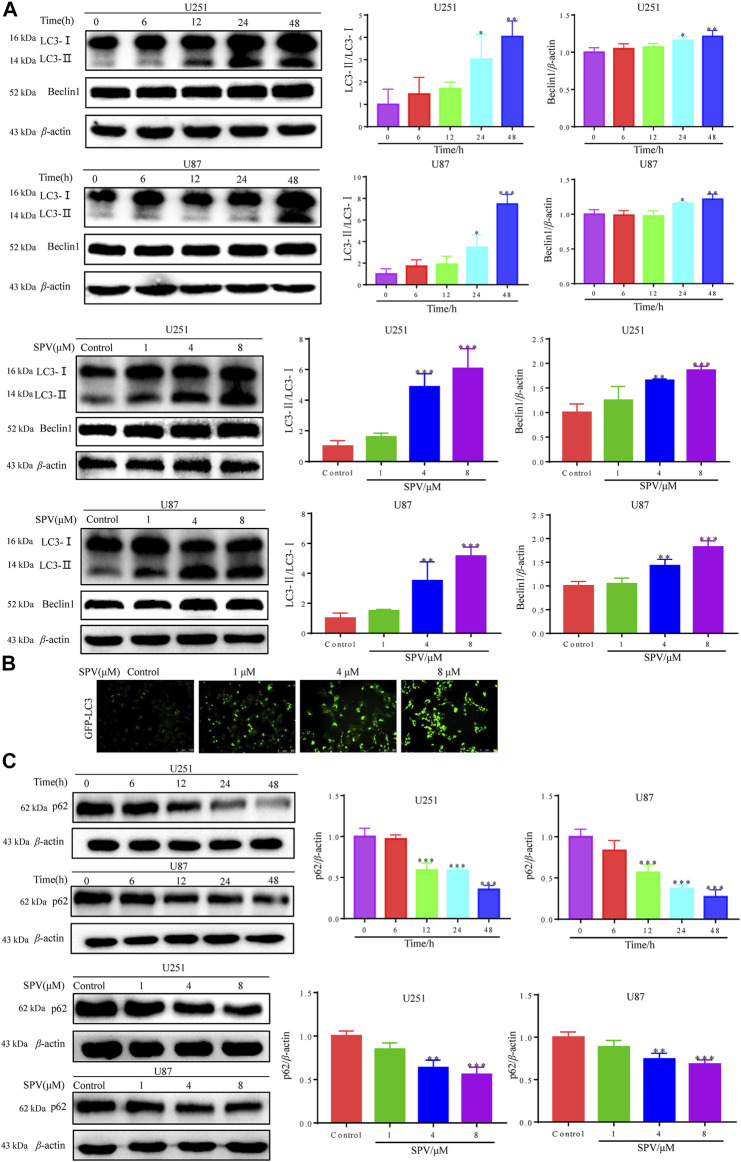
Sempervirine triggered autophagy in glioma cells. **(A)** Cells were incubated with sempervirine (4 μM) for different durations or treated with various concentrations (0, 1, 4 and 8 μM) of sempervirine for 48 h. Western blotting for autophagy-associated protein LC3 and Beclin-1. **(B)** LC3 puncta formation was measured by immunofluorescence analysis of U251 cells treated with various concentrations (0, 1, 4 and 8 μM) of sempervirine for 48 h. The images were captured using a microscope. Scale bars = 100 μm. **(C)** The autophagy-related proteins p62 was determined by Western blotting. Data are presented as the Mean ± SD (*n* = 3). *p < 0.05, **p < 0.01 and ***p < 0.001 compared with the control group.

To determine whether sempervirine induced autophagy in U251 and U87 cells, we detected the expression of autophagy biomarkers, such as LC3B and Beclin-1. The results of Western blot analysis indicated that sempervirine induced the formation of auto-phagosomes in a dose- and time-dependent manner (Figure 5A). LC3 puncta formation was measured by immunofluorescence analysis, and results indicated that the marker for autophagy increased in cells (Figure 5B). We used autophagic flux to further evaluate sempervirine-induced autophagy.

We detected changes in sempervirine-induced autophagic flux by tracking the p62 protein level. This protein binds to LC3, thereby targeting the autophagosome to promote protein clearance. A decrease in p62 protein levels was positively correlated with autophagic activity. Sempervirine significantly reduced p62 protein levels in cells in a dose- and time-dependent manner, as shown in (Figure 5C). This finding confirms that the promotion of autophagic flux occurred.

**Figure 6:**
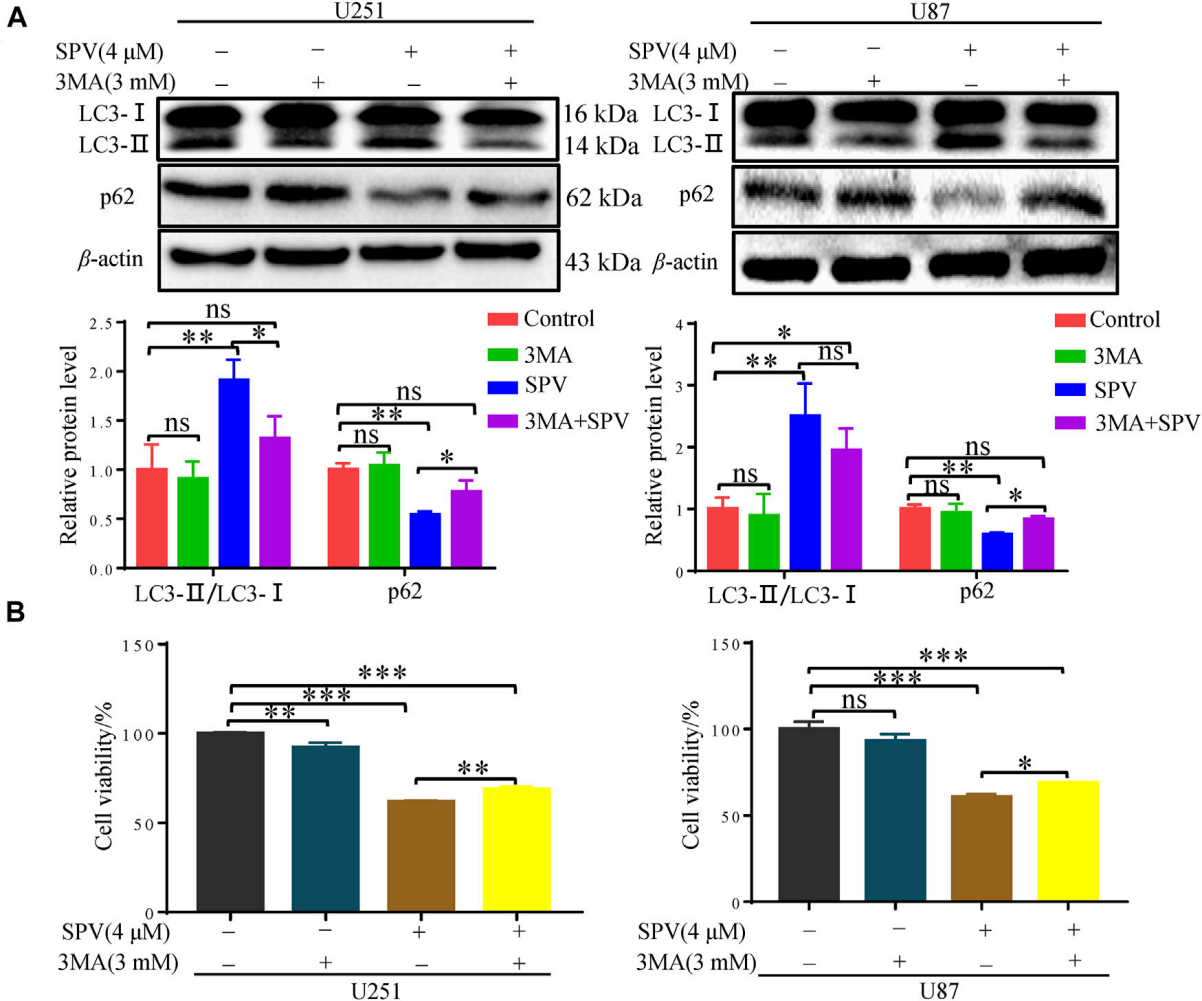
Sempervirine induces autophagic death of glioma cells. **(A)** 3-MA (3 mM) was added to cells 2 h before sempervirine treatment. After 48 h, Western blotting for autophagy-associated protein LC3 and p62. **(B)** Cell viability was determined. 3-MA was added to cells 2 h before sempervirine treatment. Then, cells treated with sempervirine for 48 h. Cell viability was determined by MTT. Data are presented as the Mean ± *SD* (*n* = 3). *p < 0.05, **p < 0.001 and ***p < 0.001 compared with the control group.

To further evaluate the occurrence of autophagy flux, we co-treated cells with sempervirine (4 μM) and autophagy inhibitor, 3-MA, which blocks the upstream steps of the process. A Western blotting assay was used to detect the expression of key proteins in autophagy, when 3-MA was used alone, obvious changes were not observed in the expression of the autophagy-related proteins LC3 and p62 compared with the control. However, 3-MA could reduce the expression of LC3B and increase the expression of p62 in the presence of sempervirine compared with the sempervirine group (Figure 6A). To verify whether sempervirine-induced cellular autophagy plays a key role in cytotoxicity, we co-treated cells with sempervirine (4 μM) and autophagy inhibitor 3-MA. Compared with the group treated with sempervirine, the co-incubation of cells with sempervirine and 3-MA increased cell survival (Figure 6B). This finding confirmed that sempervirine induced autophagic death of glioma cells.

### Sempervirine Induces Autophagic Cell Death and Apoptosis *via* AKT/mTOR Signal Pathway

**Figure 7:**
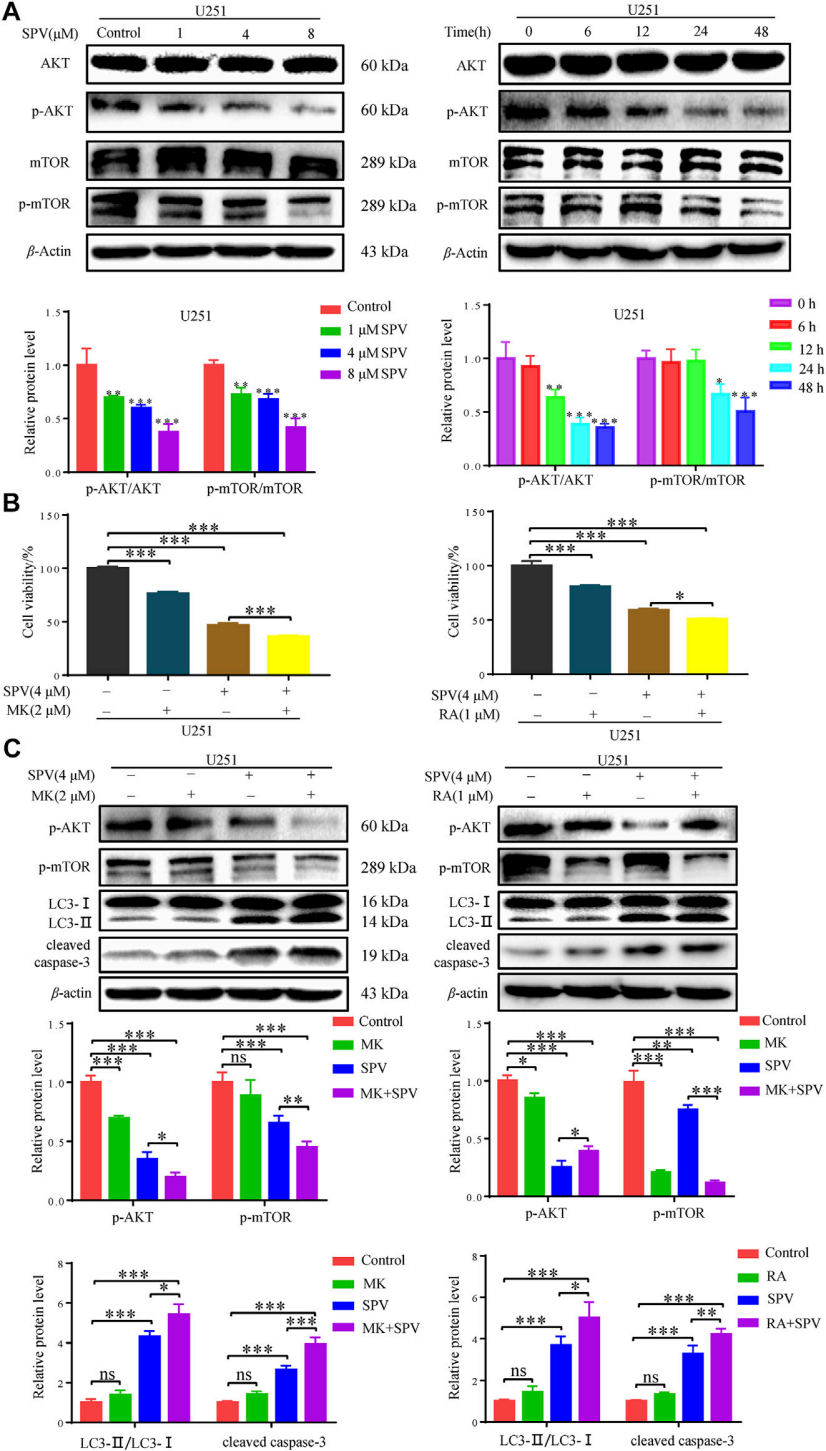
Sempervirine induced apoptosis and autophagy by inhibiting the AKT/mTOR pathway. **(A)** U251 cells were treated with various concentrations (0, 1, 4 and 8 μM) of sempervirine or incubated with sempervirine (4 μM) for different durations for 48 h. Western blotting for the level of p-AKT, AKT, p-mTOR, mTOR. **(B)** Cell viability was determined. MK (2 μM) or RA (1 μM) was added to cells 2 h before sempervirine treatment. Then, cells treated with sempervirine for 48 h. Cell viability was determined by MTT. **(C)** MK (2 μM) or RA (1 μM) was added to cells 2 h before sempervirine treatment. After 48 h, the protein level of p-AKT, p-mTOR, LC3 and cleaved caspase-3 were determined by Western blotting. Data are presented as the Mean ± SD (*n* = 3). *p < 0.05, **p < 0.01 and ***p < 0.001 compared with the control group.

To determine whether sempervirine-induced cellular responses were associated with the AKT/mTOR signaling pathway, we also examined the related proteins. Western blot analysis did not show significant changes in AKT and mTOR expressions, while phosphorylated Akt (Ser473) and phosphorylated mTOR expressions decreased. The protein expression decreased in a dose- and time-dependent manner (Figure 7A).

To determine whether sempervirine induced cell autophagy and apoptosis through the Akt/mTOR signaling pathway, further investigations were performed by using MK (MK2206, Akt inhibitor) and Ra (Rapamycin, mTOR inhibitor). As shown in (Figure 7B), in the case of cells co-treated with MK or Ra and sempervirine, cell viability was significantly lower than that observed with sempervirine treatment alone. The Western blot analysis results showed that MK and Ra further increased the expression levels of apoptosis- and autophagy-related proteins in the presence of sempervirine (Figure 7C). In summary, sempervirine induced apoptosis and autophagy by activating and blocking the Akt/mTOR signaling pathway.

### Sempervirine Inhibits the Growth of U251 in the Nude Mice Xenografted Model

**Figure 8:**
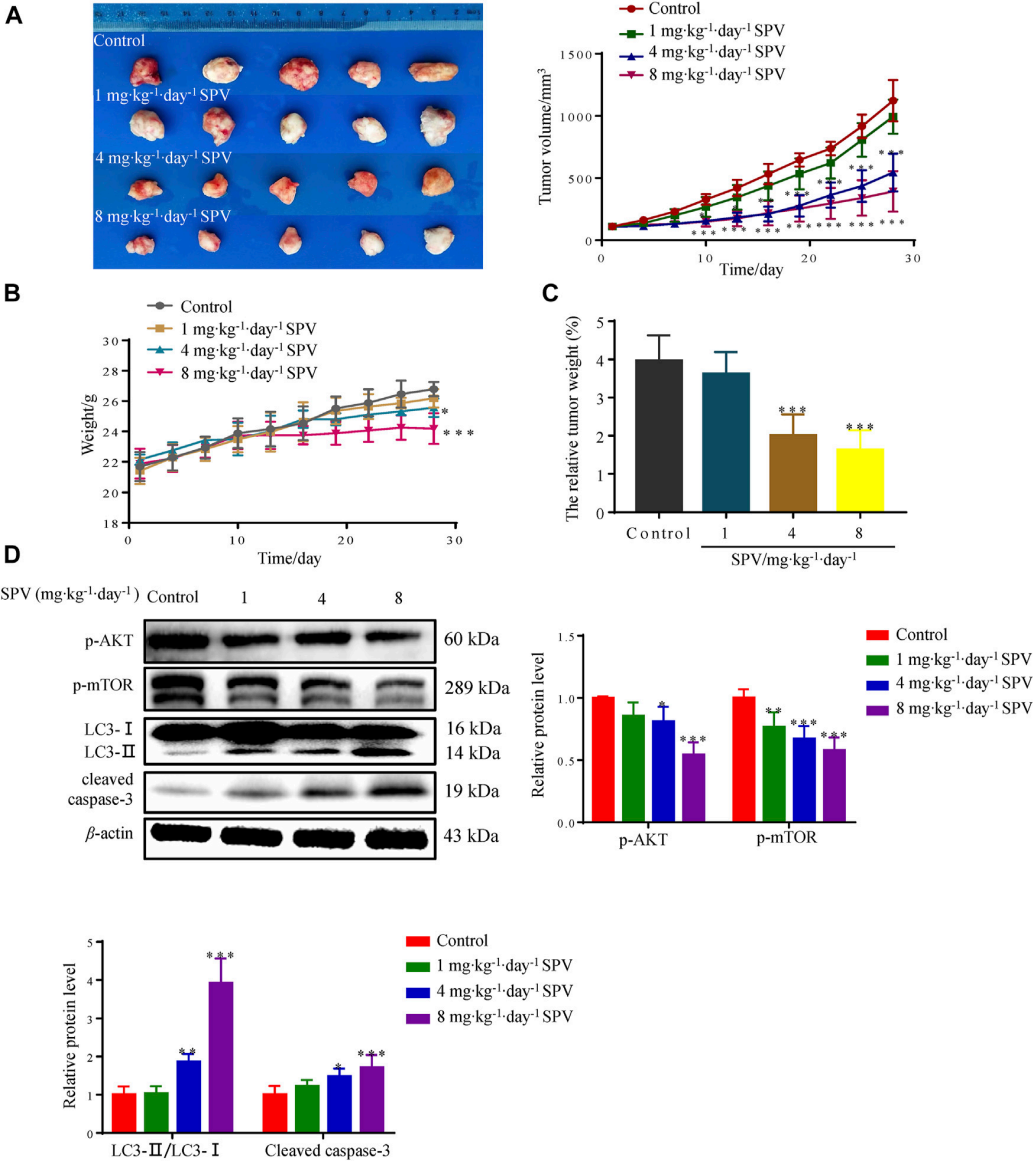
Sempervirine can inhibit the growth of U251 in the nude mice xenografted model. **(A)** The image of sempervirine inhibiting the growth of glioma (U251) cell transplant tumor with sempervirine concentration of 0, 1, 4 and 8 mg∙kg^−1^∙day^−1^ after 28 days. And the tumor volume changes in four groups with sempervirine concentration of 0, 1, 4 and 8 mg∙kg^−1^∙day^−1^ after 28 days. **(B)** The change of nude mice weight between four groups with sempervirine concentration of 0, 1, 4 and 8 mg∙kg^−1^∙day^−1^ after 28 days. **(C)** The relative tumor weight difference in four groups with sempervirine concentration of 0, 1, 4 and 8 mg∙kg^−1^∙day^−1^ after 28 days. **(D)** The protein level of p-AKT, p-mTOR, LC3 and cleaved caspase-3 in the tumor were determined by Western blotting. Data are presented as the Mean ± SD (*n* = 5). *p < 0.05, **p < 0.01 and ***p < 0.001 compared with the control group.

To further test whether sempervirine can inhibit the growth of glioma cells *in vivo*, the anti-tumor activity of sempervirine were investigated using the nude mice xenografted model. Sempervirine could significantly inhibit the growth of U251 cell transplant tumor (Figure 8A). However, the weight of nude mice in the 4 and 8 mg∙kg^−1^∙day^−1^ of sempervirine group also decreased (Figure 8B). Therefore, we adjusted the tumor weight data according to the actual weight of nude mice. We found that the intraperitoneal injection of 4 and 8 mg∙kg^−1^∙day^−1^ of sempervirine can also inhibit U251 cell tumor growth in the xenograft nude mice, with the inhibition rates being 49.46 and 58.93%, respectively. (Figure 8C). Western blot analysis showed that sempervirine significantly promoted LC3-I and LC3-II transformation and the expression of cleaved caspae-3 proteins. Western blot analysis showed that sempervirine inhibited AKT/mTOR signaling pathways in tumor tissues (Figure 8D).

## Discussion

*G. elegans* Benth. is a world-famous poisonous plant and is applied to the clinical treatment of tumors in China. Certain effect was achieved. However, because the toxic and therapeutic doses of *G. elegans* Benth were similar, its clinical application is limited. Research on the effective and low toxicity monomer of *G. elegans* Benth. has become popular in recent years.

When alkaloids or monomer was separately applied to glioma cells, sempervirine showed its anti-cancer effect. Therefore, this study aimed to explore the inhibitory effect of sempervirine on glioma cells *in vitro* and *in vivo* and its mechanism.

First, the normal cell toxicity of sempervirine and acute toxicity in mice were determined. Sempervirine at 1–8 μM had no cytotoxic effect on normal cells (3T3-L1 and THP-1 cells) and on the mice given 2 g∙kg^−1^ via intragastric administration of sempervirine. Secondly, the obvious inhibitory effect of sempervirine on glioma cells was found *in vitro*. Results of the nude mouse U251 cell transplantation tumor *in vivo* experiment showed that sempervirine at 4 and 8 mg∙kg^−1^∙day^−1^ doses could inhibit the formation of tumor. However, the weight of nude mice in the high-dose group also decreased, which is different from our experiments on normal mice. This may be caused by the difference in tolerance of immunodeficient nude mice and normal mice to sempervirine. As a result, significant antitumor effects of sempervirine were found compared with other natural compounds reported in literatures. The activity of sempervirine was significantly better than those of most natural compounds.

For example, literature has shown the berberine resistance of U251 at concentrations ranging from 50 to 100 μM (Tong et al., 2019). Aloperine, an alkaloid isolated from Sophora, can promote apoptosis of U251 cells, and the effective concentrations range from 500 to 1,000 μM (Xu et al., 2019). Resveratrol at concentrations ranging from 25 to 200 μM induced proliferation inhibition and apoptosis in U251 cells (Jiang et al., 2005). Celastrol at different concentrations ranging from 0.3 to 10 μM had inhibitory effects on U251 cells (reduced to 0.135–4.506 μg∙mL^−1^); however, its acute toxicity limits its application (Liu et al., 2019). The effective concentration of phloretin against U251 ranged from 50 to 100 μM (Hoa et al., 2007). Kukoamine A can effectively inhibit U251 at concentrations of 40–80 μM (Wang et al., 2016). The effective concentration of temozolomide, a commonly used chemotherapy drug in clinic, reaches 50–100 μM (Kil et al., 2008). In this study, the effective anti-glioma activity of sempervirine was significantly higher than that of most natural compounds reported, with IC50 of 4.981 μM (equivalent to 1.361 μg∙mL^−1^ by weight).

From the perspective of cell proliferation inhibition, the smooth operation of tumor cell cycle is a necessary condition for cell proliferation. Interference during this process can prevent cell proliferation. The G2/M cell transition period is the most important detection point for cell cycle growth, differentiation, and proliferation (Galbraith et al., 2019). Therefore, tumor cell cycle arrest is among the common mechanisms of anti-tumor proliferation (Suski et al., 2021). From the perspective of cell cycle, this study further clarified the mechanism underlying sempervirine’s inhibition of the proliferation of glioma cells (U251 and U87). This study used flow cytometry to detect the effect of sempervirine on the cell cycle of U251 and U87, respectively. Sempervirine could significantly inhibit the survival ability of glioma cells and induce G2/M phase progression arrest in U251 and U87 cells. CDK1 is the core factor of the G2/M phase conduction network, and cyclin B1 is a specific cyclin in the G2/M phase. The level of cyclin B1 gradually increased in the S and G2 phases and reached its peak in the G2/M phase. It disappeared during the transition of late mitosis. It has been reported that inhibiting the activity of CDK1 and cyclin B complex can block cells in G2/M phase (Stark and Taylor, 2006). There are also reports that cyclin B1 expression decreased when G2/M phase arrest was induced in tumor cells (Raffoul et al., 2006). The expression of cyclin B1 increased when G2/M phase arrest was induced (Wu et al., 2004), which may possibly be related to the blocked degradation of cyclin B1 protein when CDK1 activity was inhibited, thereby increasing the expression of cyclin B1 (Hershko, 1999). In the present study, sempervirine inhibited the activity of CDK1 and increased the expression of cyclin B1, we thus conjectured that sempervirine might affect CDK1-Cyclin-B kinase activity of glioma cells, and resulted in distinct cell cycle perturbation.

Cell cycle arrest and reduced cell viability can mediate apoptosis (Bruna Pucci, 2000). Sempervirine could block glioma cells in the G2/M phase, and G2/M cycle arrest was the main pathway to induce cell apoptosis. Thus, we also examined whether sempervirine could induce glioma cell apoptosis. Cell apoptosis is cell death by gene regulation. There are four main ways of participating in cell apoptosis; increased activation of caspase protein caused subsequent related protein degradation of the cascade; the activation of caspase 3 protein is the key to cell apoptosis (Deegan et al., 2014). In addition, as an important negative molecule regulating apoptosis, Bcl-2 can bind with pro-apoptotic proteins, leading to dysregulation of apoptosis and promoting the cell’s escape from apoptosis. The activation of the important pro-apoptotic protein Bax can lead to the opening of the mitochondrial permeability transporter pore, causing the release of cytochrome C (Cyt C) and finally causing apoptosis (Rodriguez et al., 2011). In the present study, Annexin V-FITC/PI staining showed that with increasing sempervirine concentration, the apoptosis rate of U251 and U87 cells increased gradually. At the protein level, sempervirine can regulate the expression of Bax/Bcl-2 and activate caspase-3 protein. In this study, we investigated RIP1, a key factor related to cell necrosis (Khan, et al., 2014), and found that sempervirine has no obvious effect on the RIP1 (Supplementary Figure S1), this suggested that sempervirine mainly induces apoptosis rather than necrosis. In addition, cells pretreated with the pan-cysteine aspartase inhibitor z-VAD-FMK showed the significantly reduced cytotoxic effect of sempervirine. z-VAD-FMK could reverse the activation of apoptotic executive protein caspase-3 induced by sempervirine, thereby confirming that sempervirine can indeed promote apoptosis. Thus, our results on apoptosis are consistent with the conventional view that sempervirine stimulates the caspase-dependent apoptosis of cancer cells. However, we found that the z-VAD-FMK does not fully recover caspase 3 cleavage in U251, which appears to be different between two cell lines.

Cell apoptosis and autophagy in biochemical pathways or morphology have significant differences, but a growing number of studies showed that these two types of programmed death are not independent. They can be activated by multiple stress stimuli, can share multiple regulatory molecules, and even coordinate with each other. They are interrelated and mutually regulated (Cerutti and Ridley, 2017; Gloushankova et al., 2017). When cells receive autophagy inductive signals, the expression of autophagy regulatory proteins in cells changes accordingly. Among them, the conversion of LC3 and the expression of p62 and Beclin-1 are the criteria for observing whether autophagy occurs (Galluzzi and Green, 2019). (1) Beclin 1 is a key target protein for regulating autophagy activity. It can initiate autophagy in cells and indirectly mediate other autophagy proteins to regulate the formation of phagocytes in cells, thus participating in the formation and maturation of autophagosomes. Its expression intensity is related to autophagy activity (Vega-Rubín-De-Celis et al., 2020). The experimental results showed that the expression of Beclin 1 increased with increasing sempervirine concentration, indicating that sempervirine can induce autophagy and increase autophagic activity through Beclin 1. (2) When autophagy was initiated, cytoplasmic LC3 enzymatically decomposed a small segment of polypeptide to form LC3-I, which combined with phosphatidylethanolamine (PE) on the membrane surface to form autophagosome membrane type (i.e., LC3-II), and the ratio of LC3-II/LC3-I increased (Hill et al., 2019; Ho and Gorski, 2019; Poillet-Perez and White, 2019). P62 can be used as a receptor to bind to ubiquitinated proteins and promote the clearance of ubiquitinated proteins, resulting in a decreased expression of p62. Therefore, p62 can be used as one of the key indicators reflecting autophagy activity (Islam et al., 2018). Western blot showed that the cells are interfered with by different concentrations of sempervirine. The expression of autophagy protein LC3-II increased with increasing drug concentration, and the ratio of LC3-II/LC3-I also showed an obvious rising trend, thereby confirming that autophagy was promoted. In addition, we examined the expression of autophagy receptor protein p62. The expression of autophagy receptor protein p62 was greatly reduced, suggesting that sempervirine could increase the autophagic flux of glioma cells. The pretreatment of cells with the early autophagy inhibitor 3-MA could significantly counteract its cytotoxic effect, and it was found that 3-MA could reverse the ratio of LC3-II/LC3-I and the expression of autophagy receptor protein p62, which further illustrated that sempervirine increases cells autophagy flux and leads to autophagic cell death.

After elucidating that sempervirine induced apoptosis and autophagy of glioma cells, this study continued to explore the mechanism underlying sempervirine-induced programmed death of glioma cells. A study has shown that sempervirine can induce cell death through the p53 pathway in testicular germ cell tumors. This suggested that sempervirine may regulate the p53 gene in tumor cells. However, when we detect the p53 protein of U87 cells treated with sempervirine, we found that sempervirine has no obvious effect on the p53 signaling pathway (Supplementary Figure S1), which indicates that sempervirine may not exert anti-glioma cell effect through the p53 pathway. The mTOR-mediated pathway is one of the important signaling pathways in the regulation of autophagy and apoptosis (Barangi et al., 2019). Inhibition of the mTOR signaling pathway induces autophagy, disrupts autophagic flux, and leads to the accumulation of autophagosomes (Cina et al., 2012). Thus, these findings support our conclusion that inhibition of mTOR phosphorylation promotes apoptosis and autophagy in sempervirine-treated glioma cells. The inhibition of this pathway is currently considered as a potential target for the treatment of tumors. The inhibitors of key components involved in this pathway, such as AKT and mTOR, have been widely explored (Alzahrani, 2019). In our study, Western blot results showed that the expression of Akt and mTOR phosphorylation level decreased significantly when cells treated with sempervirine. Our study also showed that using sempervirine in combination with Akt inhibitor (MK) or mTOR inhibitor (RA), the survival rate of glioma U251 cells was lower than when they were treated with sempervirine alone. Western blot analysis showed that sempervirine combined with MK or RA increased the expression levels of apoptosis and autophagy-related proteins in U251 cells. The above results suggested that sempervirine triggered autophagy and apoptosis, which may be mediated through the inhibition of the Akt/mTOR signaling pathway. Sempervirine apparently acted as a dual inhibitor of AKT and mTOR.

To investigate whether sempervirine also had anti-glioma activity *in vivo*, we established the nude mice xenotransplantation U251 cell tumor experiment. The results showed that significant antitumor effects were observed after sempervirine treatment at doses of 4 and 8 mg/kg. In addition, the Western blot analysis revealed a significant increase in the level of cleaved caspase-3 and LC3-II, whereas Akt and mTOR phosphorylation levels were significantly decreased.

## Conclusion

Sempervirine had a significant anti-glioma cellular effect, which was associated with sempervirine-induced cellular G2/M phase arrest and blockade of the Akt/mTOR signaling pathway, thereby triggering apoptosis and cellular autophagy. The results of this study also suggested the potential role for sempervirine in glioma treatment and provided insight into its activity.

## Data Availability

The original contributions presented in the study are included in the article/Supplementary Material, further inquiries can be directed to the corresponding authors.
