# Supplementary material for: Sempervirine Mediates Autophagy and Apoptosis via the Akt/mTOR Signaling Pathways in Glioma Cells
Source: Front Pharmacol. 2021 Nov 30;12:770667. doi: 10.3389/fphar.2021.770667 (PMC8670093; doi:10.3389/fphar.2021.770667)
Supplement: Supplementary file 1 [file DataSheet1.docx]

***Supplementary Information***

1. Supplementary Figure 1

**Supplementary Figure 1** Cells were treated with sempervirine (0, 1, 4 and 8 μM) for 48 h. (A) The necrosis-related protein RIP1 in U251 cells was determined by Western blotting. (B) After 48 hours, the levels of p53 and p-p53 in U87 cells were analyzed by Western blot.

1. Supporting cell cycle distribution for Figure. 2A

1. Supporting western blot for Figure. 2B

1. Supporting Annexin V-FITC/PI staining for Figure. 3A

1. Supporting western blot for Figure. 3B

1. Supporting western blot for Figure. 4A

1. Supporting western blot for Figure. 5A 5C

1. Supporting immunofluorescence for Figure. 5B

1. Supporting western blot for Figure. 6A

1. Supporting western blot for Figure. 7A

1. Supporting western blot for Figure. 7C

1. Supporting western blot for Figure. 8D
